# Synthesis and In Vitro Screening of Novel Heterocyclic β-d-Gluco- and β-d-Galactoconjugates as Butyrylcholinesterase Inhibitors

**DOI:** 10.3390/molecules24152833

**Published:** 2019-08-04

**Authors:** Krešimir Baumann, Lorena Kordić, Marko Močibob, Goran Šinko, Srđanka Tomić

**Affiliations:** 1 Department of Chemistry, Faculty of Science, University of Zagreb, HR-10001 Zagreb, Croatia; 2 Biochemistry and Organic Analytical Chemistry Unit, Institute for Medical Research and Occupational Health, P.O. Box 291, HR-10001 Zagreb, Croatia

**Keywords:** anticholinesterase, butyrylcholinesterase, glucose, galactose, heterocycles

## Abstract

The development of selective butyrylcholinesterase (BChE) inhibitors may improve the treatment of Alzheimer’s disease by increasing lower synaptic levels of the neurotransmitter acetylcholine, which is hydrolysed by acetylcholinesterase, as well as by overexpressed BChE. An increase in the synaptic levels of acetylcholine leads to normal cholinergic neurotransmission and improved cognitive functions. A series of 14 novel heterocyclic β-d-gluco- and β-d-galactoconjugates were designed and screened for inhibitory activity against BChE. In the kinetic studies, 4 out of 14 compounds showed an inhibitory effect towards BChE, with benzimidazolium and 1-benzylbenzimidazolium substituted β-d-gluco- and β-d-galacto-derivatives in a 10–50 micromolar range. The analysis performed by molecular modelling indicated key residues of the BChE active site, which contributed to a higher affinity toward the selected compounds. Sugar moiety in the inhibitor should enable better blood–brain barrier permeability, and thus increase bioavailability in the central nervous system of these compounds.

## 1. Introduction

**Figure 1:**
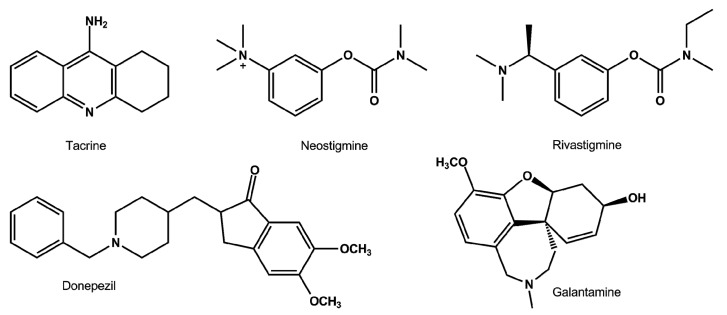
Structures of cholinesterase inhibitors used as therapeutics in Alzheimer’s disease.

Alzheimer’s disease (AD) is a chronic and progressive age-related neurological disorder, and the most common form of dementia in the industrialised part of the world. AD is a sum of genetic, physiological, and environmental factors, which affect the risk of cognitive and neurodegenerative manifestations in patients [1]. The development of AD is related to several abnormal elements, such as the aggregation of neurofibrillary tangles (NFTs), senile, tau and β-amyloid plaques, and, additionally, exposure to aluminium and brain inflammation [2]. Genetic factors that play a significant role in AD progression are associated with the brain-derived neurotrophic factor (BDNF) and butyrylcholinesterase gene (BCHE) [3]. AD is characterized by low levels of the neurotransmitter acetylcholine (ACh), due to the decreased rate of its biosynthesis [4]. It has been reported that cholinesterases, particularly butyrylcholinesterase (BChE, EC 3.1.1.8.), are associated with the pathogenesis and progression of AD [5,6]. BChE transforms the normal brain amyloid precursor protein to the β-amyloid protein, which then deposits and forms β-amyloid plaques. Neurofibrillary tangles and β-amyloid plaques accumulate in brain cells, resulting in the loss of cholinergic neurons [1,7]. Thus, BChE inhibitors may have a greater role in the treatment of AD in the future. Though a cure has not yet been found, cholinesterase inhibitors (ChEI) have been used as an effective palliative treatment for AD symptoms. The currently used ChEI therapeutics for the treatment of mild to moderate AD are neostigmine (Prostigmin^®^, Roche), rivastigmine (Exelon^®^, Novartis), donepezil (Aricept^®^, Pfizer), and galantamine (Razadyne^®^ or Reminyl^®^, Janssen), all of which act as ChEI (Figure 1) [8].

Studies of anti-ChEs show different strategies in the development of new molecules as potential drugs for the treatment of Alzheimer’s disease. Different scaffolds were recently developed or isolated and tested for anticholinesterase activity, including quinoxaline tacrine derivate [9], piperazine, piperidine, morpholinecarbodithioate derivatives [10], and the natural compound glucosinalbin, 4-hydroxybenzyl glucosinolate, isolated from *Bunias erucago* [11].

In a recent study, a structure–activity relationship analysis for a large number of AChE inhibitors was performed, quantifying several in silico parameters and inhibition potency [12]. Ligands with a higher number of sp^2^ hybridised atoms showed greater AChE inhibition potency. Some of these ligands are also active toward BChE [13,14,15,16]. Furthermore, compounds with potentially favourable motif sugar moiety or charged species (quaternary ammonium group) have shown increased bioavailability and are easily transported across cell membranes (e.g., through the blood–brain barrier) or by facilitative sugar transporters [6,17].

**Scheme 1:**
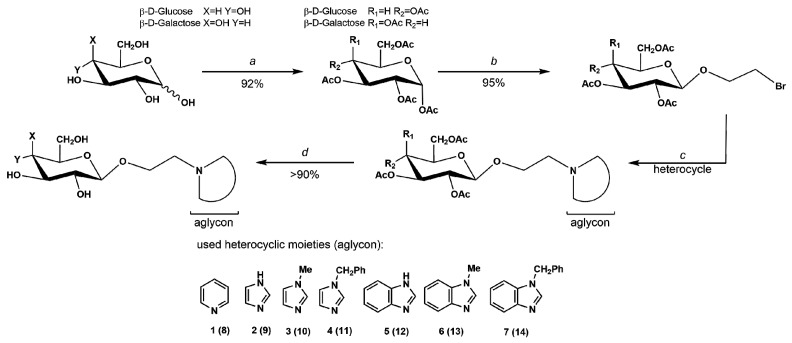
Synthetic pathway to target glucose/galactose heterocyclic derivatives. (a) (CH_3_CO)_2_O, I_2_, 5–10 min, 25 °C; (b) HOCH_2_CH_2_Br, BF_3_∙Et_2_O, dry dichloromethane, N_2_, 0 °C; (c) dry dimethylformamide, 24 h, 100 °C; (d) NaOCH_3_/CH_3_OH, 25 °C, 60 min.

Considering these facts, 14 novel heterocyclic derivatives of β-d-glucose and β-d-galactose have been synthesized and evaluated for their inhibitory activity against BChE (Scheme 1). The heterocyclic moieties used in the design of these novel compounds were pyridinium, imidazolium, and benzimidazolium derivatives, containing sp^2^ hybridised atoms, found in many natural compounds and therapeutics with diverse biological activities. The idea of introducing a C2 spacer between the glycon moiety and heterocyclic moiety was to reduce the steric hindrance effect, allowing the molecules more flexibility (increased number of conformations) and better accommodation within the BChE active site.

## 2. Results and Discussion

### 2.1. Preparation of Compounds

The preparation of the target compounds was conducted in four steps. Taking into account all synthetic steps and the desired stereochemistry of the target compounds, we chose the ester protecting group due to its key role as a neighbouring participating group in the preparation of glycosides. There are several procedures described in the literature for the acetylation of the sugar moiety, differing in the use of catalysts, solvents, and activators [18,19]. The most commonly used are pyridine, sodium acetate, ZnCl_2_, or HClO_4_. Herein, we used a fast and more environmentally friendly method of sugar acetylation, employing a catalytic amount of iodine as an activator of acetic anhydride [20].

Several glycosylation procedures have been reported and new, improved methods are constantly developed [21]. The three main reactions of introducing aglycon to a sugar moiety are the direct, Koenigs–Knorr, and trichloroacetimidate methods [21]. The introduction of a C2 spacer was conducted in the second step, by attaching 2-bromoethanol to the anomeric C-atom of peracetylated d-pyranose, using the direct method of *O*-glycosidation, in the presence of BF_3_·Et_2_O as an activator. A neighbouring equatorial acetyl group at the sugar’s C-2 atom leads to 1,2-trans glycoside, since the nucleophilic attack of alcohol at the anomeric C-atom is controlled by the steric hindrance of the acyloxonium ion intermediate [21]. The heterocyclic compounds (pyridine, imidazole, benzimidazole, and their 1-methyl and 1-benzyl derivatives) were attached to the previously prepared β-d-glucoside or β-d-galactoside. Keeping in mind the p*K*_a_ values of the used heterocyclic compounds and the solubility of the reactants, reactions with heterocycles were carried out in polar aprotic dimethylformamide (DMF) as a solvent.

Finally, three deprotection methods were tested. First, by using dibutyltin oxide (DBTO) in dry methanol, second by using dry methanol with type 4Å molecular sieves, and lastly, the Zemplén method, by adding sodium methoxide in dry methanol [22,23,24,25]. The Zemplén method showed to be fast and efficient even with a catalytic amount of sodium methoxide and had fewer byproducts despite the fact that methoxide is a hard nucleophile capable of attacking and destroying a previously formed glycoside bond.

### 2.2. Kinetic Measurements-Inhibition

**Figure 2:**
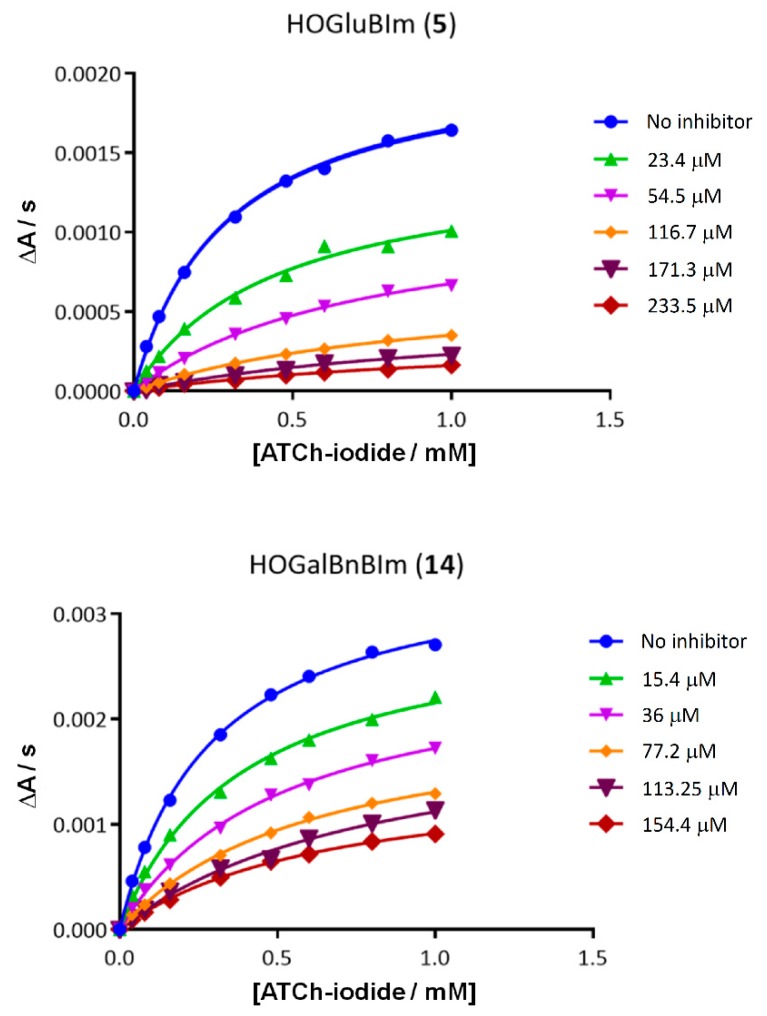
Concentration-dependent inhibition of horse serum butyrylcholinesterase (BChE) by glucoconjugate **5** and galactoconjugate **14**. The graphs represent the inhibition of BChE activity at different concentrations of acetylthiocholine as a substrate. Nonlinear regression analysis was used to determine the inhibition constant (*K*_i_) and parameter α by Equation (1).

The ability of the studied heterocyclic glycoconjugates to inhibit BChE is summarised in Table 1. Out of the 14 tested heterocyclic glycoconjugates, four compounds showed inhibition potency in the micromolar range towards equine BChE (Figure 2). Specifically, they can be structurally related to their glycon moiety as two glucoconjugates (**5**, **7**) and two galactoconjugates (**12**, **14**), or on their aglycon moiety as two benzimidazolium tertiary amines (**5**, **12**) and two N-benzylbenzimidazolium quaternary salts (**7**, **14**). Compounds **5** and **12**, and **7** and **14** can be treated as stereoisomers with the inversion of the chiral centre configuration at the sugar C-4 atom.

The results of the kinetic measurements revealed that the tested compounds act as mixed inhibitors of BChE, showing both competitive and noncompetitive inhibition with moderate inhibition potency. According to these results, the tested compounds can bind to free enzymes, as well to the Michaelis complex formed between the enzyme and substrate (ATCh). Parameter α > 1 indicates a reduction in the BChE Michaelis complex affinity towards compounds, due to the presence of a substrate within the BChE active site. An increase in the value of a parameter α confirms indirectly which residues of the active site stabilise the binding of an inhibitor in a complex with a free enzyme also involved in interactions with the substrate molecule. Usually, those were residues of the choline binding site Trp82 and Tyr332 [26].

Compounds **7** and **14** had an α of about 5, indicating a 5-fold lower affinity of BChE Michaelis complex compared to free enzymes. Interestingly, compound **12** had the lowest α value, about 2, and also the highest constant of inhibition (*K*_i_). This indicates that **12** is more likely to bind outside of the substrate binding site, and therefore the presence of a substrate in the active site does not greatly affect the affinity of the BChE Michaelis complex.

Other types of natural compounds with multiple hydroxyl groups are flavonoids, and their effect on BChE was reported in the literature [27]. The flavonoids galangin, kaempferol, quercetin, myricetin, fisetin, apigenin, luteolin, and rutin, reversibly inhibited BChE. The inhibition potency of the flavonoids was attributed to their chemical structure, i.e., the number of OH groups and their position on the phenyl ring. Galangin was the most potent inhibitor, with a *K*_i_ = (6.9 ± 2.2) µM, while rutin was the weakest one, with *K*_i_ = (501 ± 105) µM [27]. Interestingly, in the structure of rutin, a derivate of galangin, one can find two fused pyranosyl sugars via a 1–6 β bond. It was shown that the sugar moiety constitutes a steric constraint for the accommodation of rutin to the BChE active site, resulting in a lower inhibition potency. In this study, compound **5** was the most potent one, with a 30-fold higher affinity of BChE than that for rutin. This confirms that the proper selection of a sugar aromatic substituent positively increases the inhibition potency of derivatised pyranosyl sugars toward BChE. Gobec and co-workers showed in a recent study that tryptophan-derived BChE inhibitors have potential as lead compounds for symptomatic therapy against Alzheimer’s disease, with passive diffusion of the blood–brain barrier [28]. The tryptophan indol ring is similar to the benzimidazole moiety used in compounds **5** and **12**, moreover, glycon moiety should enable active transport over the blood–brain barrier. Their tryptophan-derived inhibitors showed more potency if an additional amine was present in the inhibitor molecule. Kuča and co-workers used a different approach, creating heterodimers of tacrine and tryptophan as multi-target agents with the potential to treat Alzheimer’s disease [29]. These compounds were also predicted to cross the blood–brain barrier via passive diffusion. Taking this into account, a modification in aromatic moiety that would be complementary with the BChE aromatic residues that align the active site gorge would increase the inhibition potency of aromatic pyranosyl sugars toward BChE.

### 2.3. Molecular Modelling

**Figure 3:**
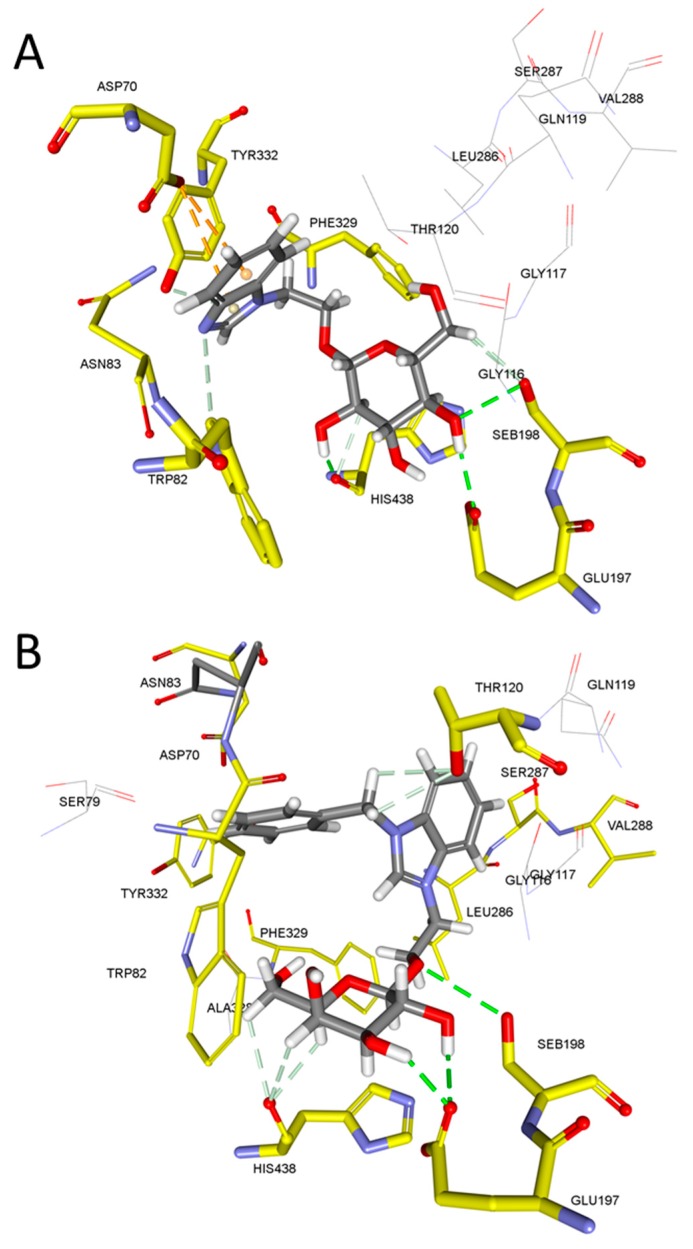
Docked conformation of tested compounds in the active site of the BChE (PDB: 2PM8). Top-ranked conformation of compound **5** (**A**). Top-ranked conformation of compound **14** (**B**). Non-covalent interactions are shown as green or light green dashed lines (H-bonds) and orange dashed lines (π interactions). Hydrogen atoms of residues are omitted for better visibility, oxygen is red, nitrogen is blue, and carbon is grey or yellow.

For a better understanding of BChE interactions with pyranosyl sugars, we performed molecular modelling. The results of molecular modelling for compound **5** are shown in Figure 3A. Compound **5** was the most potent inhibitor in this set, although the difference in *K*_i_ values for **5**, **7**, and **14** were not significant enough to be rationalized with molecular docking with respect to a specific interaction between the ligand and active site residues.

Compound **5,** with its hydroxyl groups, forms multiple hydrogen bonds with active site gorge residues: Glu197, Ser198, and His438. These residues constitute a BChE catalytic site and this is the reason why parameter α is increased for the binding of **5** to the Michaelis complex—the presence of substrate (ATCh) resulted in a reduction in its affinity.

The benzimidazole radical is analogue with a tryptophan indole ring where one nitrogen atom is replaced with a carbon atom. In the crystal structure of BChE, tryptophans are very often involved in an interaction with other aromatic residues. Interestingly, BChE has a network of aromatic residues in the active site. This network, rich in π-electrons, includes Trp82, the residue involved in the mechanism of substrate hydrolysis, and the surrounding four additional residues Tyr128, Tyr332, Trp430, and Tyr440 [30]. Close to this network are two also relevant aromatic residues: Trp231 and Phe329. The same residues can be found in the active site of AChE: Trp86, Tyr133, Tyr341, Trp439, Tyr449, Trp236, and Phe338. The main site of tacrine (see Figure 1) interaction with BChE is Trp82 [26] and with AChE Trp86 [31], a conserved residue that forms a choline binding site. All these aromatic residues may contribute to the stabilisation of the benzimidazole or benzylbenzimidazole part of tested compounds. Figure 3A depicts the interaction of benzimidazole with Trp82, Tyr332, and a close proximity to Phe329 is visible. There is also a π-anion interaction of benzimidazole with Asp70, a residue of BChE substrate peripheral binding site.

The binding mode of compound **14** is shown in Figure 3B. The major difference between **5** and **14** was the replacement of a hydrogen atom with a benzyl substituent at the benzimidazole ring. This change enlarged the aromatic radical attached via C2-spacer to the sugar. Additionally, a positive charge occurred at the N3 nitrogen. Compound **14,** like compound **5,** formed multiple hydrogen bonds with active site gorge residues: Glu197, Ser198, and His438. The sugar moiety was positioned close to Trp82. There was a T-shaped orientation between the benzyl group and Trp332, and a carbon–hydrogen bond with Thr120. The C2-spacer was in close contact with Phe329, the distance being 4.23 Å. Benzimidazole was located in the pocket formed by Gly117 from an oxyanion hole, and Val288 from an acyl binding site.

Since the central nervous system (CNS) active drugs show specific physicochemical properties, we compared a set of properties to those that exist for known AD drugs (see Figure 1). As can be seen, the properties for compounds **1**–**7** were within the recommended values for the CNS active drugs, as well as donepezil (*K*_i_, AChE = 5.7 nM), galantamine (*K*_i_, AChE = 0.5 µM), and rivastigmine (*K*_i_, AChE = 0.7 µM) [12]. Compounds **1**–**7** showed a higher number of hydrogen bonds donors due to the presence of hydroxyl groups in the sugar moiety. For the same reason, the polar surface area was also larger than that for CNS active drugs. Compounds **8**–**14** were stereoisomers with **1**–**7** and were not included in the analysis, since they shared their physicochemical properties. Table 2 lists six physicochemical properties in relation to the recommended values of CNS active drugs, which generally have lower molecular weights (MW < 450), have moderate hydrophobicity (logP < 5), have fewer hydrogen bonds donors and acceptors (HBD < 3 and HBA < 7), fewer rotatable bonds (RB < 8), and are less polar (polar surface area PSA < 70 Å2) than drugs that are not active in the CNS [32].

## 3. Materials and Methods

### 3.1. Chemicals

All reagents and solvents were used as purchased from commercial suppliers (Kemika, Zagreb, Croatia; Sigma Chemical Co., St. Louis, MO, USA) without further purification. d-glucose, d-galactose, 1,2,3,4,6-penta-*O*-acetyl-β-d-glucose, pyridine, imidazole, and benzimidazole were commercially available (Sigma Chemical Co., St. Louis, MO, USA). The procedure for the preparation of 1-methyl and 1-benzyl imidazole/benzimidazole derivatives was given in our previously published paper [33]. The reactions were monitored by thin-layer chromatography (TLC) plates, Silica Gel 60 F_254_ plates (Merck, Darmstadt, Germany). TLC plates were visualized by ultraviolet irradiation (254 nm) or by iodine fumes. Iodine promoted solvent free sugar acetylation was conducted, employing an already known procedure [19,20], as well as the introduction of an ethyl spacer in the second step by the direct method of *O*-glycosidation [21], resulting in pure 2-bromoethyl β-d-glycosides. Target molecules were prepared according to the general procedure described below. ^1^H- and ^13^C-nuclear magnetic resonance (NMR) spectra were recorded at 22 °C with a Bruker Avance III HD 400 MHz/54 mm Ascend spectrometer (Bruker Optics Inc., Billerica, MA, USA). Chemical shifts were given in ppm downfield from tetramethylsilane (TMS) as an internal standard (δ = 0.00 ppm) and coupling constants (J) in Hz. Splitting patterns were designated as s (singlet), d (doublet), dd (doublet of doublets), t (triplet), q (quartet), or m (multiplet). Atoms of glycon moiety were numerated 1–6, while atoms in aglycon moiety linking glycon with heterocycle were marked with (‘) and numbers 1 or 2, i.e., (H’-1, C’-1). Atoms in heterocycle were marked with the corresponding abbreviation (Py, Im, BIm) and numerated (HIm-1, CIm-4). Mass spectra were obtained using an Agilent 1200 series high-performance liquid chromatography (HPLC) and an Agilent 6410 Triple Quadrupole mass spectrometer equipped with an electrospray ionization source (Agilent Technologies, Lexington, MA, USA). Infrared (IR) spectra were recorded on a Fourier-transform infrared spectroscopy (FTIR) PerkinElmer Spectrum Two spectrometer (PerkinElmer, Inc., Waltham, MA, USA). The purity of the final products, tested with Agilent 1200 series HPLC, was more than 94%.

General procedure: A stirred solution of 2-bromoethyl-peracetylated-D-glycoside (0.1 mol) and an appropriate heterocyclic compound (0.15 mol) in dry dimethylformamide (0.5 mL) was heated approximately for 24 h at 100 °C under an inert argon atmosphere. The completion of the reaction was monitored by TLC (CHCl_3_:CH_3_OH = 9:1). The reaction mixture was concentrated on the rotary evaporator, and the resulting residue was purified by column chromatography on silica gel using gradient eluation (CHCl_3_:CH_3_OH = 9:1 to 100% CH_3_OH), yielding an oily syrup. To the obtained syrup dissolved in dry methanol, 25% sodium methoxide in methanol was added (~50 μL). The completion of the reaction was monitored by TLC or IR. After no reactant material or no absorption band for C=O (1750–1735 cm^−1^) was observed, the reaction mixture was concentrated under reduced pressure and the residue was purified by column chromatography on silica gel using gradient eluation (CHCl_3_:CH_3_OH = 9:1 to 100% CH_3_OH).

*N-[2-(β-d-glucopyranozyloxy)ethyl]pyridinium bromide* (**1**) Colourless syrup, quaternary salt, (0.1159 g, Yield 89%, ${\left[\alpha \right]}_{D}^{25}$ = −48° (c 0.1, MeOH)); IR (KBr) ν/cm^−1^: 3392, 2955–2856 (C-H), 1636 (C=N), 1076–1041; ^1^H-NMR (400 MHz, CD_3_OD) δ/ppm: 8.89 (d, 2H, HPy-2,6), 8.74 (t, 2H, HPy-3,5), 8.22 (t, 1H, HPy-4), 4.89 (m, 2H), 4.3 (d, 1H, HGlu-1), 4.17–4.09 (m, 3H), 4.0 (m, 1H), 3.78 (m, 2H), 3.68–3.63 (m, 2H), 3.6–3.46 (m, 3H); ^13^C-NMR (101 MHz, CD_3_OD) δ/ppm: 146.1 (CPy-2,4,6), 128.4 (CPy-3,5), 111.8 (CGlu-1), 76.8, 73.8, 71.5, 68.8 (CGlu-2,3,4,5), 66.4 (CGlu-6), 62.2 (C′-2, CH2-O), 59.1 (C′-1, CH2-N+); ESI-MS (C_13_H_20_NO_6_^+^) *m*/*z*: found 286.1 (calculated 286.1).

*N1-[2-(β-d-glucopyranozyloxy)ethyl]imidazole* (**2**) Yellowish syrup, tertiary amine, (0.098 g, Yield 73%, ${\left[\alpha \right]}_{D}^{25}$ = −40° (c 0.1, MeOH)); IR (KBr) ν/cm^−1^: 3401 (O-H), 2942–2863 (C-H), 1632 (C=N), 1444, 1075; ^1^H-NMR (400 MHz, CD_3_OD) δ/ppm: 7.75 (s, 1H, HIm-2), 7.23 (s, 1H, HIm-4), 6.95 (s, 1H, HIm-5), 4.82 (s, 2H), 4.32-4.25 (m, 3H), 4.16–4.13 (m, 2H), 3.9–3.87 (m, 2H), 3.69–3.67 (m, 3H), 3.36–3.21 (m, 3H); ^13^C-NMR (101 MHz, CD_3_OD) δ/ppm: 137.6 (CIm-2), 127.2 (CIm-4), 119.8 (CIm-5) 103.1 (CGlu-1), 76.6 (CGlu-5), 73.6 (CGlu-3), 70.1 (CGlu-2), 68.6 (CGlu-4), 68.6 (CGlu-6), 61.3 (C′-2, CH2-O), 46.8 (C′-1, CH2-N+); ESI-MS (C_11_H_18_N_2_O_6_-H^+^) *m*/*z*: found 275.1 (calculated 274.1).

*N1-methyl-N3-[2-(β-d-glucopyranozyloxy)ethyl]imidazolium bromide* (**3**) Yellowish syrup, quaternary salt, (0.075 g, Yield 63%, ${\left[\alpha \right]}_{D}^{25}$ = −36° (c 0.1, MeOH)); IR (KBr) ν/cm^−1^: 3392 (O-H), 2942–2855 (C-H), 1631 (C=N), 1447, 1085; ^1^H-NMR (400 MHz, dimethyl sulfoxide (DMSO)-d6) δ/ppm: 9.07 (s, 1H, HIm-2), 7.77 (d, 1H, *J* = 2 Hz, HIm-4), 7.69 (d, 1H, *J* = 2.1 Hz, HIm-5), 5.15 (d, 1H, *J* = 1.59 Hz, HGlu-1), 5.02–4.96 (m, 2H, CH2-N+), 4.56 (t, 1H), 4.37 (t, 1H), 4.21–4.19 (d, 1H), 3.9 (t, 1H), 3.85 (s, 3H, CH3-N), 3.68 (m, 1H), 3.42 (m, 1H), 3.12 (m, 2H), 2.99 (m, 2H); ^13^C-NMR (101 MHz, DMSO-*d*_6_) δ/ppm: 137.6 (CIm-2), 123.7 (CIm-5), 123.1 (CIm-4), 103.2 (CGlu-1), 77.4, 76.9, 73.7, 70.4 (CGal-2-5), 67.4 (CGal-6), 61.5 (C′-1, CH2-N+), 49.5 (CH3-N), 36.2 (C′-2, CH2-O); ESI-MS (C_12_H_21_N_2_O_6_^+^) *m*/*z*: found 289.1 (calculated 289.1).

*N1-benzyl-N3-[2-(β-d-glucopyranozyloxy)ethyl]imidazolium bromide* (**4**) Yellowish syrup, quaternary salt, (0.056 g, Yield 58%, ${\left[\alpha \right]}_{D}^{25}$ = −39° (c 0.1, MeOH)); IR (KBr) ν/cm^−1^: 3381 (O-H), 2950–2835 (C-H), 1628 (C=N), 1447, 1077; ^1^H-NMR (400 MHz, DMSO-*d*_6_) δ/ppm: 9.36 (s, 1H, HIm-2), 7.84 (d, 2H, HIm-4,5), 7.44–7.42 (m, 5H, HBn-2-6), 5.47 (s, 2H), 5.17 (d, 1H), 5.04 (d, 1H), 4.97 (d, 1H), 4.61 (t, 2H), 4.42 (t, 1H), 4.24–4.22 (d, 1H), 4.03 (m, 1H), 3.93 (m, 1H), 3.68 (m, 1H), 3.4 (m, 1H), 3.36 (s, 1H), 3.15 (m, 1H), 3.03-2.96 (m, 2H); ^13^C-NMR (101 MHz, DMSO-*d*_6_) δ/ppm: 137.4 (CIm-2), 135.3 (CBn-1), 129.4 (CBn-3,5), 129.1 (CBn-4), 128.7 (CBn-2,6), 123.7 (CIm-5), 122.5 (CIm-4), 103.2 (CGlu-1), 77.3 (CGlu-5), 76.9 (CGlu-3), 73.7 (CGlu-2), 70.4 (CGlu-4), 67.3 (CGlu-6), 61.4 (C′-2, CH2-O), 52.2 (C′-1, CH2-N+), 49.7 (Ph-CH2-N); ESI-MS (C_18_H_25_N_2_O_6_^+^) *m*/*z*: found 365.1 (calculated 365.1).

*N1-[2-(β-d-glucopyranozyloxy)ethyl]benzimidazole* (**5**) Colourless syrup, tertiary amine, (0.063 g, Yield 63%, ${\left[\alpha \right]}_{D}^{25}$ = −42° (c 0.1, MeOH)); IR (KBr) ν/cm^−1^: 3387 (O-H), 2936–2853 (C-H), 1645 (C=N), 1474, 1073; ^1^H-NMR (400 MHz, DMSO-*d*_6_) δ/ppm: 8.27 (s, 1H), 7.67–7.64 (m, 3H), 7.27–7.18 (m, 2H), 5.12–5.04 (d, 1H), 4.57 (s, 2H), 4.46 (s, 1H), 4.25 (d, 1H), 4.05 (m, 1H), 3.91 (m, 2H), 3.75 (d, 1H), 3.41 (s, 1H), 3.18–3.11 (m, 1H), 3.07–3.02 (m, 2H); ^13^C-NMR (101 MHz, DMSO-*d*_6_) δ/ppm: 145.2 (CBIm-2), 142.7 (CBIm-9), 136.4 (CBIm-8), 123 (CBIm-5), 122.1 (CBIm-6), 119.5 (CBIm-4), 111.1 (CBIm-7), 102.4 (CGlu-1), 78.1, 76.3, 72.9, 71.2, 66.9 (CGlu-2,3,4,5,6), 61.3 (C′-2, CH2-O), 48.8 (C′-1, CH2-N); ESI-MS (C_15_H_20_N_2_O_6_-H^+^) *m*/*z*: found 325.0 (calculated 324.1). See Figures S1–S3.

*N1-methyl-N3-[2-(β-d-glucopyranozyloxy)ethyl]benzimidazolium bromide* (**6**) Yellowish syrup, quaternary salt, (0.089 g, Yield 44%, ${\left[\alpha \right]}_{D}^{25}$ = −58° (c 0.1, MeOH)); IR (KBr) ν/cm^−1^: 3393 (O-H), 2940–2837 (C-H), 1651 (C=N), 1569, 1364, 1071; ^1^H-NMR (400 MHz, DMSO-*d*_6_) δ/ppm: 8.86 (s, 1H, HBIm-2), 8.4 (m, 2H, HBIm-4,5), 7.76 (m, 2H, HBIm-6,7), 4.83 (t, 2H, CH2-N+), 4.39 (s, 3H, CH3-N), 5.02 (m, 1H, HGlu-1), 3.86-3.7 (m, 4H), 3.6-3.49 (m, 8H); ^13^C-NMR (101 MHz, DMSO-*d*_6_) δ/ppm: 141.2 (CBIm-2), 129.6 (CBIm-9), 128.2 (CBIm-8), 127.3, 126.7 (CBIm-5,6), 116.7, 111.9 (CBIm-4,7), 103.7 (CGlu-1), 75.2, 73.5, 71.7, 67.4 (CGlu-6), 61.1 (C′-1, CH2-N+), 48.5 (CH3-N), 42.8 (C′-2, CH2-O); ESI-MS (C_16_H_23_N_2_O_6_^+^) *m*/*z*: found 339.2 (calculated 339.1).

*N1-benzyl-N3-[2-(β-d-glucopyranozyloxy)ethyl]benzimidazolium bromide* (**7**) Yellowish syrup, quaternary salt, (0.117 g, Yield 39%, ${\left[\alpha \right]}_{D}^{25}$ = −52° (c 0.1, MeOH)); IR (KBr) ν/cm^−1^: 3416 (O-H), 2954–2851 (C-H), 1639 (C=N), 1473, 1077; ^1^H-NMR (400 MHz, DMSO-*d*_6_) δ/ppm: 8.7 (s, 1H, HBIm-2), 8.5 (m, 2H, HBIm-6,7), 7.7 (m, 2H, HBIm-4,5), 7.32–7.25 (m, 5H, HBn-2,3,4,5,6), 5.4 (s, 2H, Ph-CH2-N), 4.9 (m, 3H), 4.4 (m, 1H), 4.3 (m, 1H), 3.89 (d, 1H), 3.9–3.62 (m, 5H), 3.4 (m, 4H); ^13^C-NMR (101 MHz, DMSO-*d*_6_) δ/ppm: 145.9 (CBIm-2), 137.4 (CBn-1), 133.7, 131.13 (CBIm-8,9), 129.06, 128.7, 128.08, 126.8, 126.7 (CBn-2,3,4,5,6), 125.4 (CBIm-5,6), 113.5 (CBIm-4,7), 106.7 (CGlu-1), 76.2, 73.6, 71.4, 68.3 (CGlu-2,3,4,5), 64.4 (CGlu-6), 60.8 (C′-2, CH2-O), 52.6 (C′-1, CH2-N+), 49.7 (N-CH2-Ph); ESI-MS (C_22_H_27_N_2_O_6_^+^) *m*/*z*: found 415.2 (calculated 415.1). See Figures S4–S6.

*N-[2-(β-d-galactopyranozyloxy)ethyl]pyridinium bromide* (**8**) Colourless syrup, quaternary salt, (0.0849 g, Yield 69%, ${\left[\alpha \right]}_{D}^{25}$ = −30° (c 0.1, MeOH)); IR (KBr) ν/cm^−1^: 3401 (O-H), 2950–2919 (C-H), 1629 (C=N), 1488, 1383, 1088; ^1^H-NMR (400 MHz, CD_3_OD) δ/ppm: 9.11 (d, 2H, *J* = 5.79 Hz, HPy-2,6), 8.65 (t, 2H, *J_1_* = 7.73 Hz, *J_2_* = 7.73 Hz, HPy-3,5), 8.15 (t, 2H, *J1* = 7.24 Hz, *J2* = 7.24 Hz, HPy-4), 4.97–4.88 (m, 2H) 4.38–4.32 (m, 2H), 4.26–4.21 (m,1H), 4.14–4.08 (m, 1H), 3.94–3.88 (m, 3H), 3.85 (d, 1H, *J* = 1,88 Hz, HGal-1), 3.7–3.69 (d, 2H), 3.6–3.46 (m, 3H); ^13^C-NMR (101 MHz, CD_3_OD) δ/ppm: 145.7 (CPy-2,4,6), 127.85 (CPy-3,5), 103.8 (CGal-1), 75.4, 73.3, 70.8, 68.8 (CGal-2,3,4,5), 67.9 (CGal-6), 61.6 (C′-2, CH2-O), 61.1 (C′-1, CH2-N+); ESI-MS (C_13_H_20_NO_6_^+^) *m*/*z*: found 286.1 (calculated 286.1).

*N1-[2-(β-d-galactopyranozyloxy)ethyl]imidazole* (**9**) Yellowish syrup, tertiary amine, (0.1258 g, Yield 98%, ${\left[\alpha \right]}_{D}^{25}$ = −60° (c 0.1, MeOH)); IR (KBr) ν/cm^−1^: 3353 (O-H), 2938–2870 (C-H), 1631 (C=N), 1463, 1080; ^1^H-NMR (400 MHz, CD3OD) δ/ppm: 7.75 (s, 1H, HIm-2), 7.7 (s, 1H), 7.23 (t, 1H), 7.07 (d, 2H, *J* = 0.96 Hz, HIm-4,5), 6.95 (t, 1H), 4.28–4.24 (m, 3H), 4.17–4.13 (m, 1H), 3.9-3.84 (m, 2H), 3.79–3.71 (m, 3H), 3.58–3.47 (m, 3H); ^13^C-NMR (101 MHz, CD_3_OD) δ/ppm: 137.6 (CIm-2) 127.2 (CIm-4), 119.9 (CIm-5) 103.8 (CGal-1), 75.4 (CGal-5), 73.5 (CGal-3), 71.1 (CGal-2), 68.9 (CGal-4), 68.6 (CGal-6), 61.4 (C′-2, CH2-O), 46.9 (C′-1, CH2-N+); ESI-MS (C_11_H_18_N_2_O_6_-H+) *m*/*z*: found 275.1 (calculated 274.1).

*N1-methyl-N3-[2-(β-d-galactopyranozyloxy)ethyl]imidazolium bromide* (**10**) Yellowish syrup, quaternary salt, (0.0779 g, Yield 19%, ${\left[\alpha \right]}_{D}^{25}$ = −30° (c 0.1, MeOH)); IR (KBr) ν/cm^−1^: 3368 (O-H), 2948–2833 (C-H), 1628 (C=N), 1575, 1474, 1454, 1383, 1087, 1033; ^1^H-NMR (400 MHz, CDCl_3_) δ/ppm: 8.02 (s, 1H, HIm-2), 7.73 (d, 1H, *J* = 1.97 Hz, HIm-4), 7.64 (d, 1H, *J* = 1.9 Hz, HIm-5), 4.85 (d, 1H, *J* = 1.59 Hz, HGal-1), 4.55–4.52 (m, 2H, H′-2, CH2-N+), 4.13–4.07 (m, 1H), 4.01 (s, 3H, CH3-N), 3.90–3.86 (m, 2H), 3.81–3.69 (m, 3H), 3.66–3.62 (t, 2H, *J_1_* = 9.5 Hz, *J_2_* = 9.57 Hz, H′-1), 3.36 (s, 1H), 3.24–3.2 (m, 3H); ^13^C-NMR (101 MHz, CDCl_3_) δ/ppm: 137.5 (CIm-2), 127.2 (CIm-5), 119.8 (CIm-4), 103.8 (CGal-1), 75.4, 73.5, 71.0, 68.9 (CGal-2-5), 68.6 (CGal-6), 61.1 (C′-1, CH2-N+), 48.4 (CH3-N), 46.9 (C′-2, CH2-O); ESI-MS (C_12_H_21_N_2_O_6_^+^) *m*/*z*: found 353.3 (289.1 + 2CH_3_OH) (calculated 289.1).

*N1-benzyl-N3-[2-(β-d-galactopyranozyloxy)ethyl]imidazolium bromide* (**11**) Yellowish syrup, quaternary salt, (0.0117 g, Yield 25%, ${\left[\alpha \right]}_{D}^{25}$ = −30° (c 0.1, MeOH)); IR (KBr) ν/cm-1: 3391 (O-H), 2928–2890 (C-H), 1638 (C=N), 1562, 1455, 1155, 1072; ^1^H-NMR (400 MHz, CDCl_3_) δ/ppm: 7.76 (d, 1H), 7.62 (d, 1H), 7.52–7.4 (m, 6H), 5.49 (s, 2H), 4.58–4.48 (m,3H), 4.37–4.35 (d, 1H), 4.23–4.18 (m, 1H), 4.08–4.03 (m, 1H), 3.89 (d, 1H), 3.86–3.82 (m, 1H), 3.76–3.68 (m, 2H), 3.62–3.55 (m, 3H); ^13^C-NMR (101 MHz, CDCl_3_) δ/ppm: 136.8 (CIm-2), 134.0 (CBn-1), 129.1 (CBn-3,5), 128.9 (CBn-4), 128.4 (CBn-2,6), 123.3 (CIm-5), 121.8 (CIm-4), 103.7 (CGal-1), 75.4 (CGal-5), 73.4 (CGal-3), 70.9 (CGal-2), 68.9 (CGal-4), 67.4 (CGal-6), 61.2 (C′-2, CH2-O), 52.7 (C′-1, CH2-N+), 49.88 (Ph-CH2-N); ESI-MS (C_18_H_25_N_2_O_6_^+^) *m*/*z*: found 365.2 (calculated 365.1).

*N1-[2-(β-d-galactopyranozyloxy)ethyl]benzimidazole* (**12**) Yellowish syrup, tertiary amine, (0.0641g, Yield 18%, ${\left[\alpha \right]}_{D}^{25}$ = −33° (c = 0.1, MeOH)); IR (KBr) ν/cm^−1^: 3380 (O-H), 2926 (C-H), 1655 (C=N), 1499, 1292, 1075; ^1^H-NMR (400 MHz, CDCl_3_) δ/ppm: 7.75 (s, 1H, HBim-2), 7.69 (m, 2H), 7.27 (m, 2H), 4.90 (m, 2H, C′-1, CH2-N), 4.28–4.26 (m, 3H), 4.17–4.12 (m, 3H), 3.91–3.83 (m, 2H), 3.80–3.70 (m, 2H), 3.57–3.45 (m, 1H); ^13^C-NMR (101 MHz, CDCl_3_) δ/ppm: 137.57 (CBim-2), 134.93 (CBim-8,9), 127.73 (CBim-5,6), 119.86 (CBim-4,7), 103.8 (CGal-1), 75.4, 73.5, 71.0, 68.9, (CGal-2,3,4,5), 68.6 (CGal-6), 61.2 (C′-2, CH2-O), 48.4 (C′-1, CH2-N); ESI-MS (C_15_H_20_N_2_O_6_-H+) *m*/*z*: found 325.2 (calculated 324.1). See Figures S7–S9.

*N1-methyl-N3-[2-(β-d-galactopyranozyloxy)ethyl]benzimidazolium bromide* (**13**) Yellowish syrup, quaternary salt, (0.1051 g, Yield 20%, ${\left[\alpha \right]}_{D}^{25}$ = −78° (c 0.1, MeOH)); IR (KBr) ν/cm^−1^: 3413 (O-H), 2926–2860 (C-H), 1642 (C=N), 1572, 1384, 1073; ^1^H-NMR (400 MHz, CDCl_3_) δ/ppm: 8.33 (s, 1H, HBim-2), 8.1–8.07 (m, 1H), 8.0–7.99 (m, 1H), 7.77–7.74 (m, 1H), 7.3–6.69 (m, 1H), 4.86 (t, 2H, H′-1, CH2-N+), 4.23 (s, 3H, CH3-N), 4.20 (m, H′-2), 4.01-3.96 (m, 4H), 3.83-3.56 (m, 5H); ^13^C-NMR (101 MHz, CDCl_3_) δ/ppm: 144.7 (CBIm-2), 129.6 (CBIm-9), 128.2 (CBIm-8), 127.3, 126.7 (CBIm-5,6), 116.7, 111.9 (CBIm-4,7), 103.7 (CGal-1), 75.2 (CBIm-5), 73.5 (CBIm-3), 71.7 (CBIm-2), 96.0 (CBIm-4), 67.4 (CBIm-6), 61.1 (C′-1, CH2-N+), 48.5 (CH3-N), 42.8 (C′-2, CH2-O); ESI-MS (C_16_H_23_N_2_O_6_^+^) *m*/*z*: found 339.2 (calculated 339.1).

*N1-benzyl-N3-[2-(β-d-galactopyranozyloxy)ethyl]benzimidazolium bromide* (**14**) Yellowish syrup, quaternary salt, (0.1726 g, Yield 31%, ${\left[\alpha \right]}_{D}^{25}$ = −57° (c 0.1, MeOH)); IR (KBr) ν/cm^−1^: 3416 (O-H), 3054–2951 (C-H), 1629 (C=N), 1563, 1478, 1456, 1384, 1077; ^1^H-NMR (400 MHz, CDCl_3_) δ/ppm: 8.1–8.08 (d, 2H, *J* = 7.83 Hz, HBIm-6,7), 7.87–7.84 (d, 2H, *J* = 8.2 Hz, HBim-4,5), 7.38–7.7 (m, 7H, 5 HBn), 5.83 (s, 2H, Ph-CH2-N), 4.39 (d, 1H), 4.31 (m, 1H), 4.20 (m, 1H), 3.89 (d, 1H, *J* = 1.93 Hz, HGal-1), 3.83–3.55 (m, 6H), 3.36 (m, 4H); ^13^C-NMR (101 MHz, CDCl3) δ/ppm: 133.37 (CBim-2, CBn-1), 131.7, 131.13 (CBim-8,9), 129.06, 128.7, 128.08, 126.8, 126.7 (CBn-2,3,4,5,6), 113.16, 113.5 (CBIm-4,7), 103.7 (CGal-1), 75.2, 73.3, 70.9, 68.8 (CGal-2,3,4,5), 66.4 (CGal-6), 61.0 (C′-2, CH2-O), 50.6 (C′-1, CH2-N+), 47.7 (N-CH2-Ph); ESI-MS (C_22_H_27_N_2_O_6_^+^) *m*/*z*: found 415.2 (calculated 415.2). See Figures S10–S12.

### 3.2. Inhibition of BChE by Heterocyclic β-d-Glycoconjugates

The stock solutions were prepared in water or 0.1 M phosphate buffer (pH 7.4), calibrated prior to the measurements. If necessary, DMSO was added to aid the dissolution of the test compounds; the final DMSO concentration was kept under 0.5% to eliminate its influence on enzyme activity [34]. Lyophilised BChE from horse serum, acetylthiocholine iodide (ATCh), and thiol reagent 5,5′-dihiobis(2-nitrobenzoic acid) (DTNB) were purchased from Sigma–Aldrich (St. Louis, MO, USA). Lyophilised BChE was chosen over AChE from human red blood cells and BChE from human serum due to the competitive reactions with other enzymes/proteins (primary proteases and albumin) that could interfere with experimental parameters, such as enzyme kinetics, during the biochemical assay. Horse serum BChE was also chosen due to a very high sequence homology with human plasma BChE [35]. Moreover, the activity of BChE, unlike AChE, was not affected by the addition of 1–5% DMSO concentration used for compound dissolution [34]. The activity of BChE (0.08 U/mL final concentration) was assayed by the Ellman spectrophotometric method [36], using ATCh-iodide as a substrate (concentration range 0.04–1 mM), and the thiol reagent DTNB (0.3 mM, ε = 14,250 dm^3^ mol^−1^ cm^−1^; [37]) in the presence of compounds **1**–**14** (10–240 μM final concentration range).

The increase in the absorbance of the TNB anion was measured in a 0.1 M sodium phosphate buffer (pH 7.4) at 25 °C and 412 nm up to 90 s in 2 mL total volume quartz cuvettes on an AnalytikJena Specord 200 spectrophotometer (Analitik, Jena, Germany). Compound-induced ATCh-iodide nonenzymatic hydrolysis was not observed. Enzyme activity was measured at six substrate concentrations for five compound concentrations in triplicate. The experimentally obtained initial reaction rates at different substrate concentrations (ATCh) were fitted to Michaelis–Menten kinetics, wherefrom *K*_m_ and *V*_m_ were obtained, employing nonlinear regression using GraphPad Prism6 software (GraphPad, San Diego, CA, USA). The enzyme-inhibitor dissociation constant, *K*_i_, and binding affinity parameter α were evaluated from the four-parameter model of mixed inhibition related to Equation (1), using a global optimization algorithm [38]:

(1) $$v_{i}=\frac{V_{m}^{\prime }[S]}{K_{m}^{\prime }+[S]}\;V_{m}^{\prime }=V_{m}\frac{1}{1+\frac{\left[I\right]}{\alpha K_{i}}}\;K_{m}^{\prime }=K_{m}\frac{1+\frac{\left[I\right]}{K_{i}}}{1+\frac{\left[I\right]}{\alpha K_{i}}}.$$

### 3.3. Molecular Modelling

The compounds to be docked in the active site of human BChE were modelled and later minimized with the MMFF94 force field using ChemBio3D Ultra 12.0 (PerkinElmer, Inc., Waltham, MA, USA). The Discovery Studio 2017 R2 (Accelrys, San Diego, CA, USA) Dock Ligands protocol (CDOCKER) with a CHARMm force field was used for the docking study [39,40]. The model of human BChE was the crystal structure of BChE (PDB: 2PM8) and it was used as the rigid receptor [41]. The binding site within the BChE was defined by a sphere (r = 13 Å) [42]. Details about docking procedure using the CDOCKER protocol and scoring of generated ligand poses by a CHARMm energy were described previously [42].

### 3.4. In Silico Prediction of Blood–Brain Barrier Penetration

The potential of novel compounds to cross the blood–brain barrier via passive diffusion was evaluated by molecular descriptors: the polar surface area (PSA), the molecular weight (MW), the calculated logarithm of the octanol/water partition coefficient (logP), the number of hydrogen bond acceptors (HBA), the number of hydrogen bond donors (HBD), and the number of rotatable bonds (RB). Molecular descriptors were determined using the Discovery Studio 2017 R2 (BioVia/Accelrys, San Diego, CA, USA). The in silico determined results were compared to the recommendations of physicochemical properties for known central nervous system drugs [32].

## 4. Conclusions

In conclusion, a series of multifunctional heterocyclic-based sugar analogues were designed by merging two known biologically active structural moieties that can show favourable effects for counteracting the progression and pathogenesis of Alzheimer’s disease. In this study, we presented the inhibition of lyophilised equine BChE by 14 novel heterocyclic β-d-gluco- and β-d-galactoconjugates. Compounds were prepared in four synthetic steps, in good or moderate yields. The direct measurement of inhibition showed that 4 out of 14 tested compounds acted as mixed inhibitors of BChE. Compounds **5** and **14** showed good inhibition potency in the micromolar range towards BChE with *K*_i_ = (16 ± 1.6) μM and (26.2 ± 2.7) μM, respectively. Benzimidazole derivatives of pyranosyl sugars represent the structural scaffold that can serve as a lead in the development of anti-AD drugs. The introduction of a modification in the aromatic moiety that would be complementary with BChE aromatic residues that align the active site gorge would increase the inhibition potency of aromatic pyranosyl sugars toward BChE. Additionally, the introduction of another type of tertiary amines may also have positive effects on inhibition potency, as Gobec and co-workers showed [28]. A comparison of the physicochemical properties of the tested compounds in relation to the recommended values of CNS active drugs confirmed their potential in the treatment of AD.

## Figures and Tables

**Table 1:**
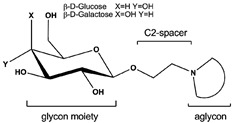
Prepared heterocyclic β-d-glycoconjugates **1**–**14** and inhibition potency toward horse serum BChE.

Compound	Sugar/Glycon	Heterocycle/Aglycon	Substitution Position on Aglycon	Concentration (μM)	*K*_i_ ± SEM (μM) ^1^	α ± SEM ^1^
**1**	D-GLUCOSE	Pyridine (Py)	N	10–140	n.d.^2^	n.d.^2^
**2**		Imidazole (Im)	N1	10–150	n.d.^2^	n.d.^2^
**3**		1-methylimidazole (MeIm)	N3	10–150	n.d.^2^	n.d.^2^
**4**		1-benzylimidazole (BnIm)	N3	10–150	n.d.^2^	n.d.^2^
**5**		Benzimidazole (BIm)	N1	20–240	16 ± 1.6	3.3 ± 0.7
**6**		1-methylbenzimidazole (MeBIm)	N3	10–120	n.d.^2^	n.d.^2^
**7**		1-benzylbenzimidazole (BnBIm)	N3	10–110	52 ± 3.0	5.1 ± 0.8
**8**	D-GALACTOSE	Pyridine (Py)	N	10–110	n.d.^2^	n.d.^2^
**9**		Imidazole (Im)	N1	15–200	n.d.^2^	n.d.^2^
**10**		1-methylimidazole (MeIm)	N3	10–160	n.d.^2^	n.d.^2^
**11**		1-benzylimidazole (BnIm)	N3	15–160	n.d.^2^	n.d.^2^
**12**		Benzimidazole (BIm)	N1	15–170	782 ± 306	2.1 ± 1.7
**13**		1-methylbenzimidazole (MeBIm)	N3	10–140	n.d.^2^	n.d.^2^
**14**		1-benzylbenzimidazole (BnBIm)	N3	15–160	26 ± 2.7	5.2 ± 1.4

^1^ Inhibition parameters were obtained by nonlinear regression using Equation (1). Values are the mean ±standard error of the mean (SEM) of three independent measurements. ^2^ Not determined.

**Table 2:** Comparison of physicochemical properties for compounds **1**–**7** with recommended values for the central nervous system (CNS) active drugs. Known anti-AD (Alzheimer’s disease) drugs donepezil, galantamine, and rivastigmine are given for comparison.

	Physicochemical Properties					
Compound	MW ^1^	logP ^2^	HBD ^3^	HBA ^4^	RB ^5^	PSA ^6^
**1**	286	–0.85	4	2	5	107
**2**	275	–1.23	5	2	5	122
**3**	289	–1.02	4	2	5	112
**4**	365	0.56	4	2	7	112
**5**	325	0.26	5	2	5	122
**6**	339	0.47	4	2	5	112
**7**	415	2.05	4	2	7	112
Donepezil	379	4.57	0	3	6	39
Galantamine	287	1.44	1	2	1	42
Rivastigmine	250	2.60	0	2	5	33
The recommended values for CNS drugs [32]	450	5	3	7	8	70

^1^ MW—molecular weight; ^2^ logP—lipophilicity coefficient; ^3^ HBD—the number of hydrogen bonds donors; ^4^ HBA—the number of hydrogen bonds acceptors; ^5^ RB—rotatable bonds; ^6^ PSA—the polar surface area.

## Supplementary Materials

The following are available online, ESI-MS, ^1^H and ^13^C NMR spectra of active compounds (**5**, **7**, **12** and **14**; Figures S1–S12).
