# Supplementary material for: Synthesis and In Vitro Screening of Novel Heterocyclic β-d-Gluco- and β-d-Galactoconjugates as Butyrylcholinesterase Inhibitors
Source: Molecules. 2019 Aug 4;24(15):2833. doi: 10.3390/molecules24152833 (PMC6695897; doi:10.3390/molecules24152833)
Supplement: Supplementary file 1 [file molecules-24-02833-s001.pdf]

Synthesis and In Vitro Screening of Novel Heterocyclic β-D-Gluco- and β-D-Galactoconjugates as Butyrylcholinesterase Inhibitors

Krešimir Baumann [1], Lorena Kordić [1], Marko Močibob [1], Goran Šinko [2,*] and Srđanka Tomić [1]

[1] Department of Chemistry, Faculty of Science, University of Zagreb, HR-10001 Zagreb, Croatia
[2] Biochemistry and Organic Analytical Chemistry Unit, Institute for Medical Research and Occupational Health, POB 291, HR-10001 Zagreb, Croatia
* Correspondence: gsinko@imi.hr; Tel.: +385-1-4682-500

Content

S1. ESI-MS, ^{1}H and ^{13}C NMR spectra of active compounds 2

*N1-[2-(β-D-glucopyranozyloxy)ethyl]benzimidazole (**5**) 2*

*N1-benzyl-N3-[2-(β-D-glucopyranozyloxy)ethyl]benzimidazolium bromide (**7**)..................................3*

*N1-[2-(β-D-galactopyranozyloxy)ethyl]benzimidazole (**12**)..5*

*N1-benzyl-N3-[2-(β-D-galactopyranozyloxy)ethyl]benzimidazolium bromide (**14**)...............................6*

S1. ESI-MS, ^{1}H and ^{13}C NMR spectra of active compounds

*N1-[2-(β-D-glucopyranozyloxy)ethyl]benzimidazole (**5**)*

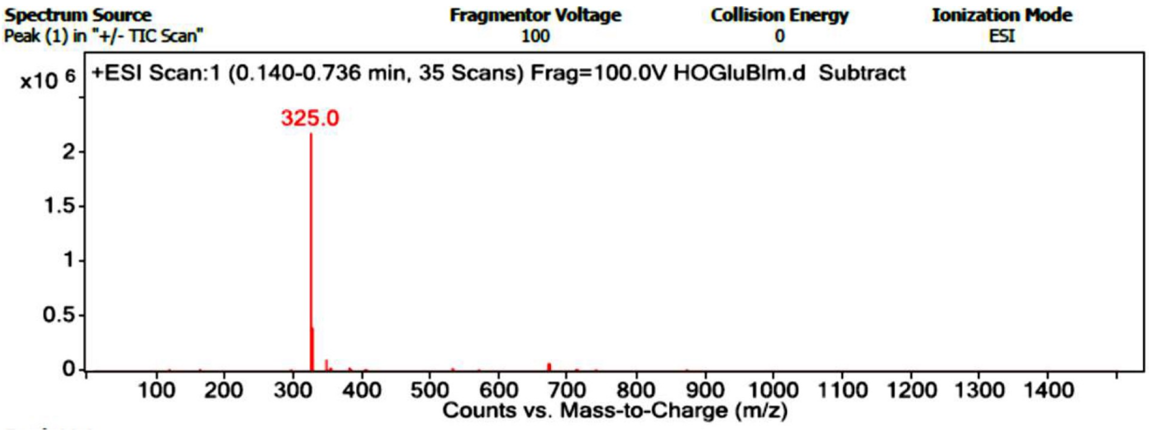

Figure S1. ESI-MS spectra of compound (**5**)

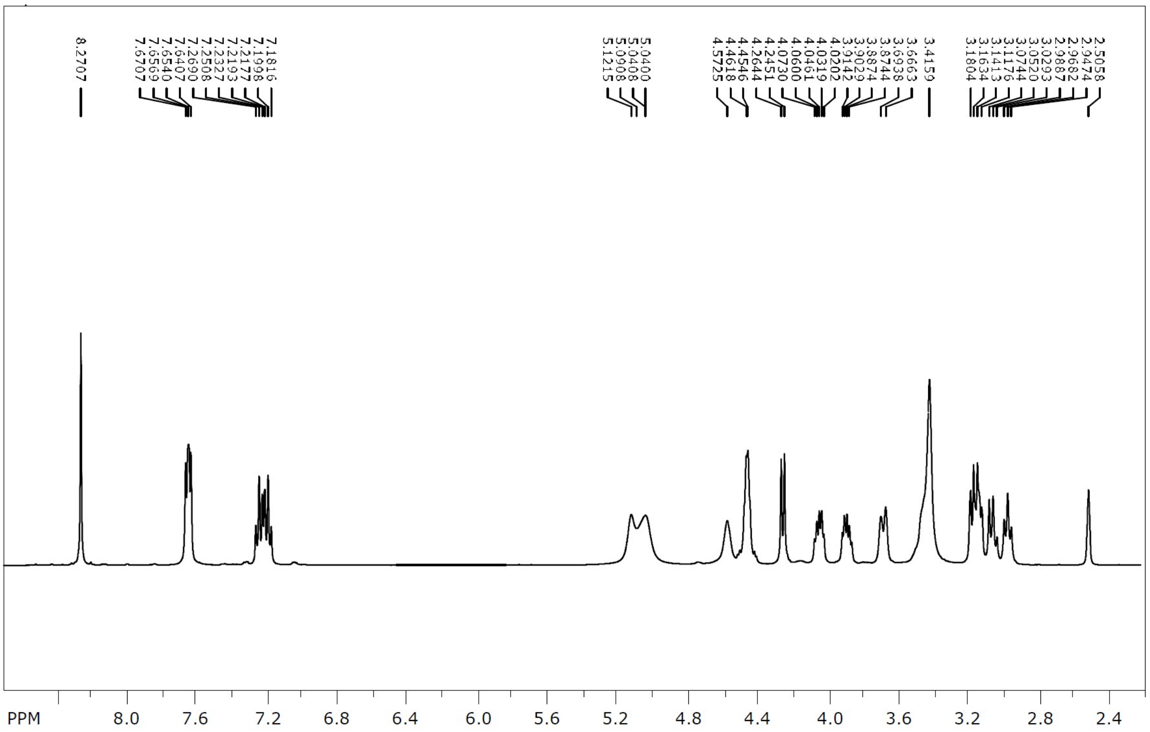

Figure S2. ^{1}H NMR spectra of compound (**5**)

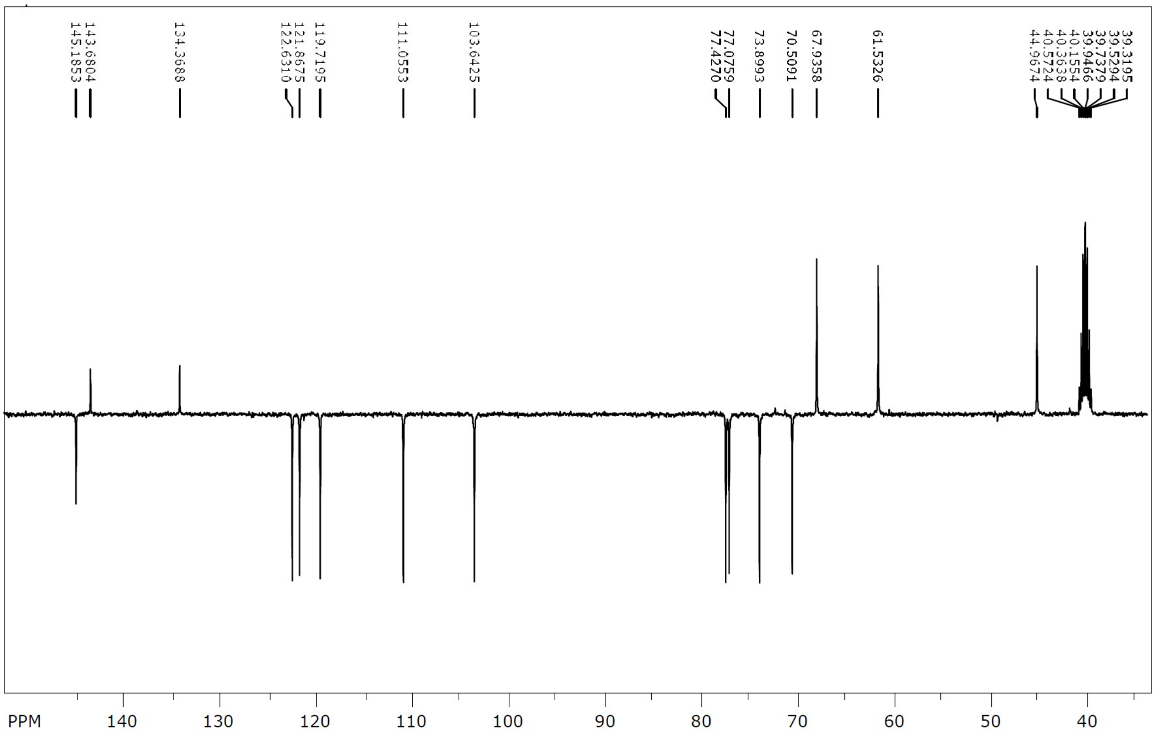

Figure S3. ^{13}C NMR spectra of compound (**5**)

N1-benzyl-N3-[2-(β-D-glucopyranozyloxy)ethyl]benzimidazolium bromide (**7**)

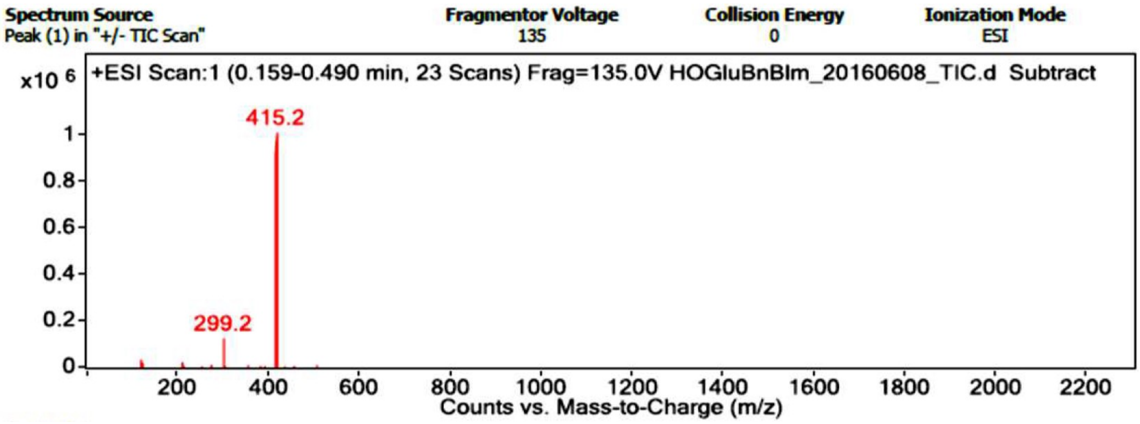

Figure S4. ESI-MS spectra of compound (**7**)

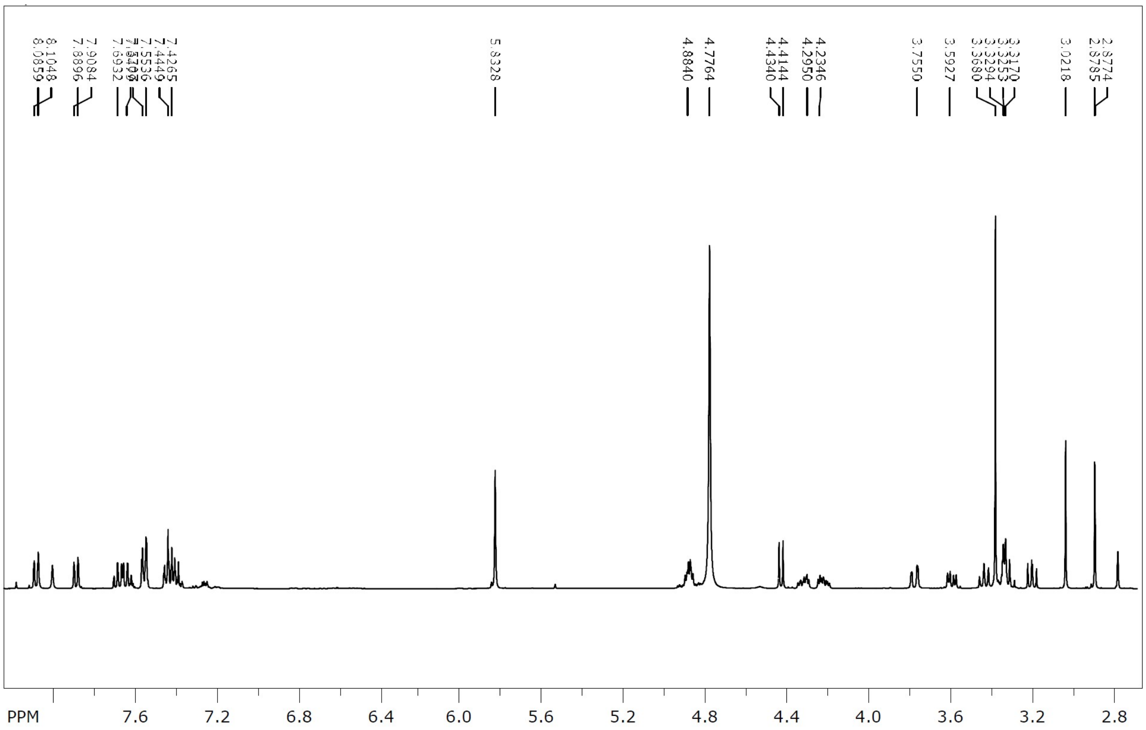

Figure S5. ^{1}H NMR spectra of compound (**7**)

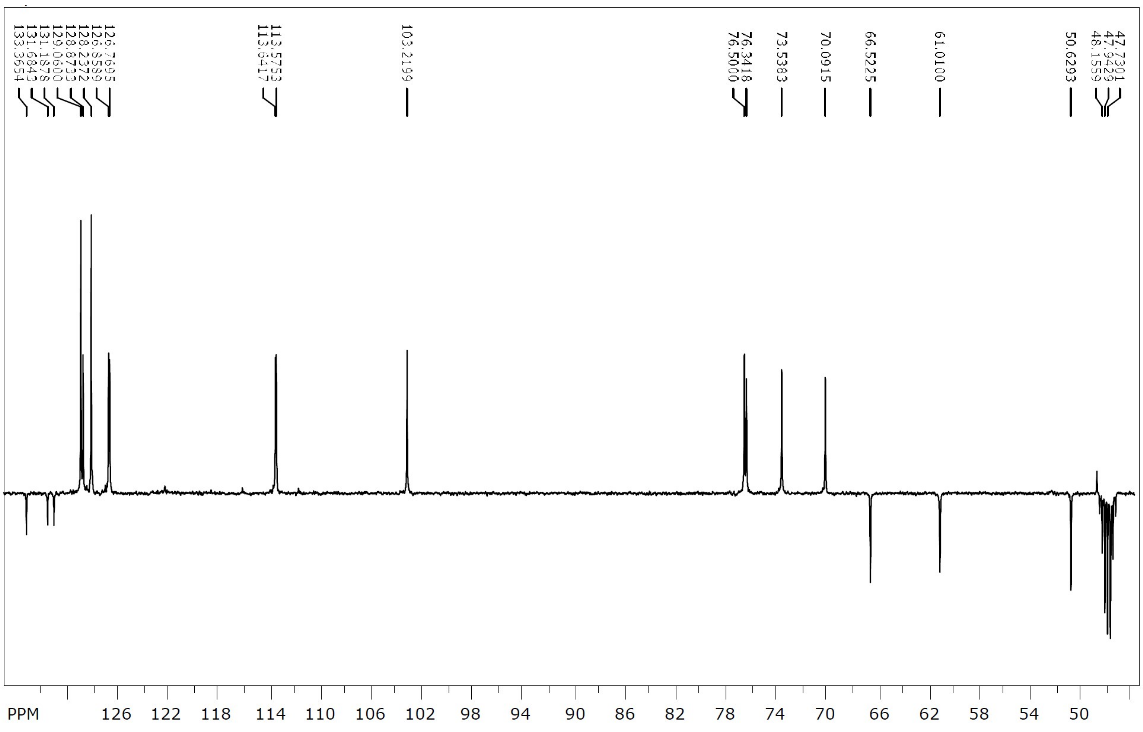

Figure S6. ^{13}C NMR spectra of compound (**7**)

*N1-[2-(β-D-galactopyranozyloxy)ethyl]benzimidazole (**12**)*

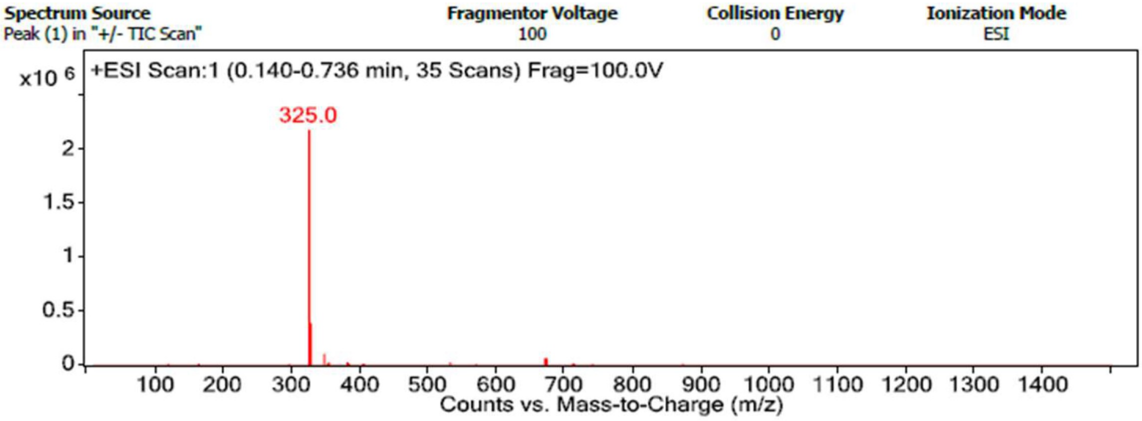

Figure S7. ESI-MS spectra of compound (**12**)

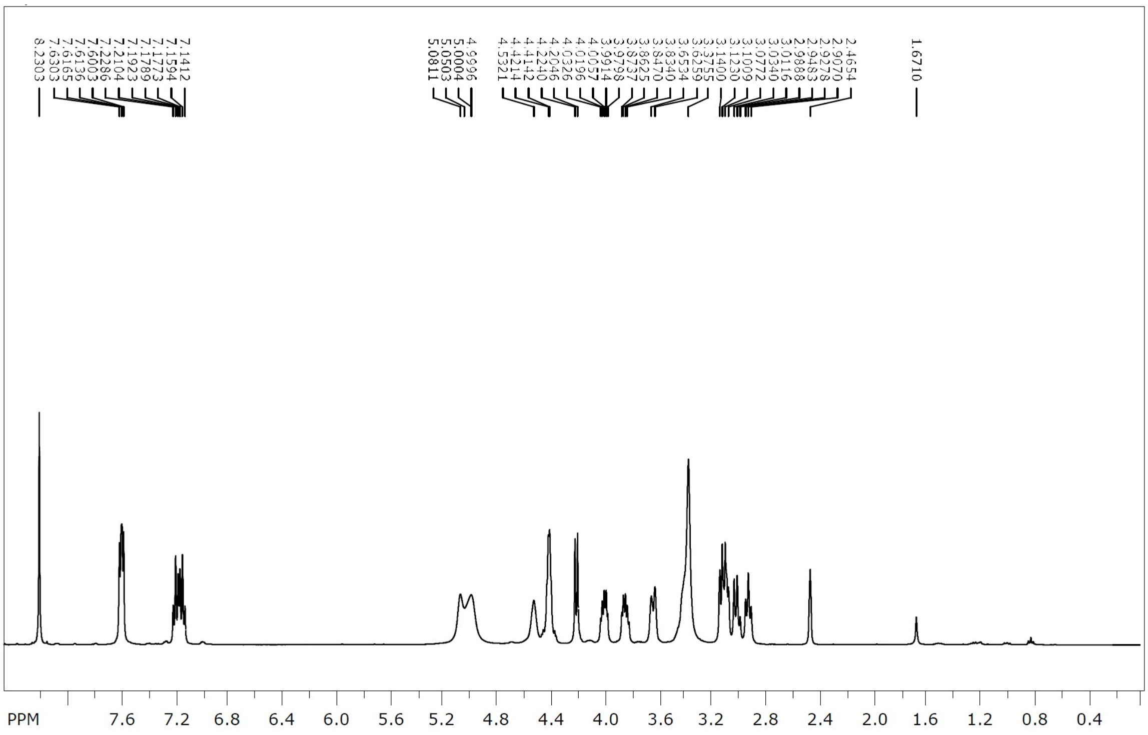

Figure S8. ^{1}H NMR spectra of compound (**12**)

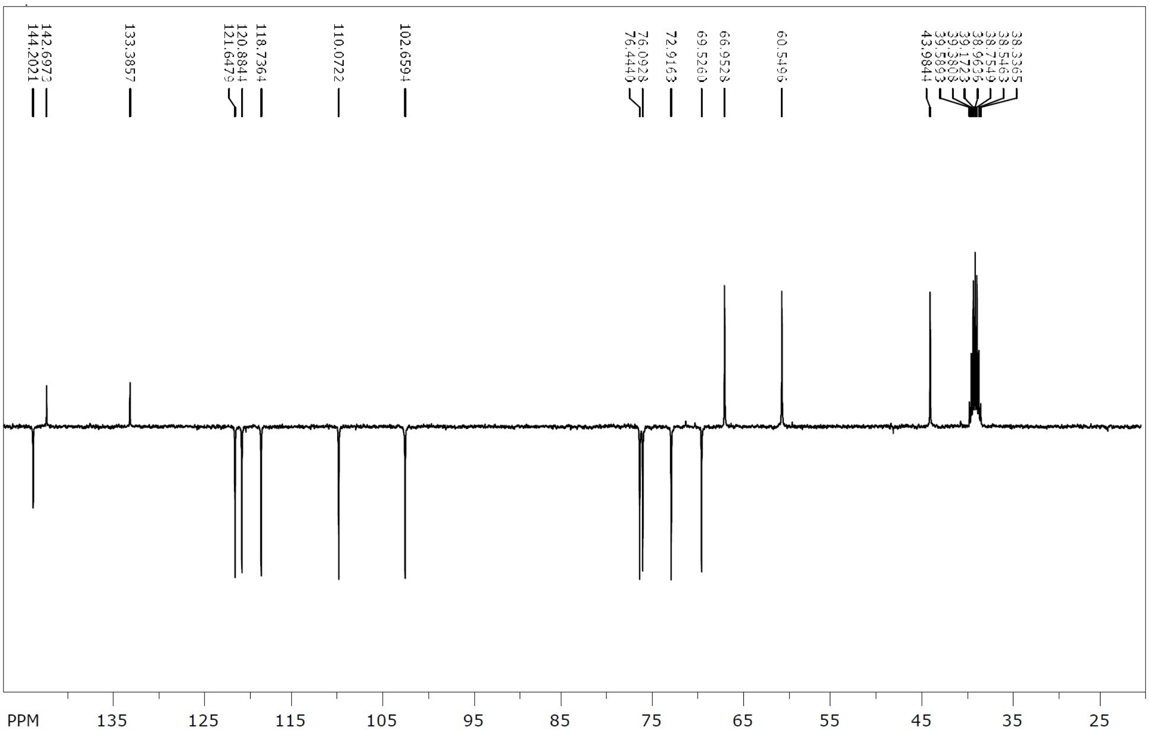

Figure S9. ^{13}C NMR spectra of compound (**12**)

N1-benzyl-N3-[2-(β-D-galactopyranozyloxy)ethyl]benzimidazolium bromide (***14***)

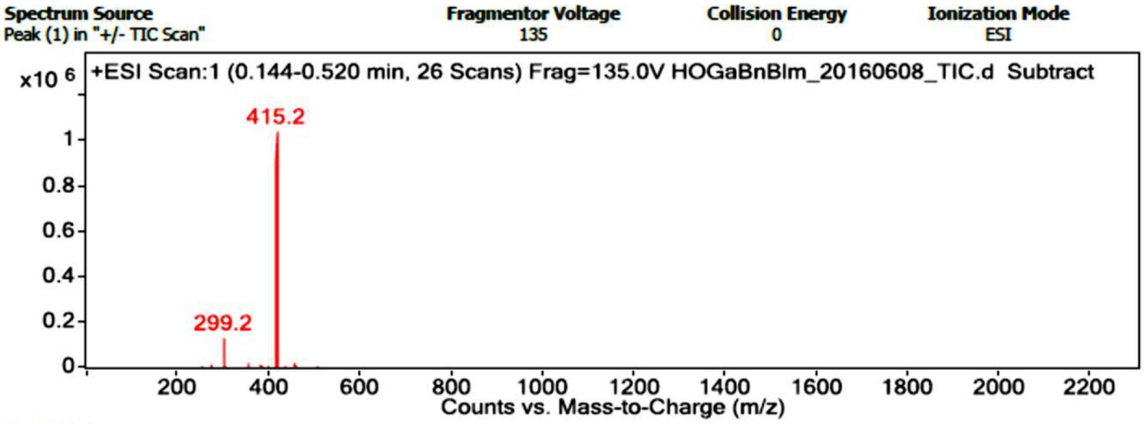

Figure S10. ESI-MS spectra of compound (**14**)

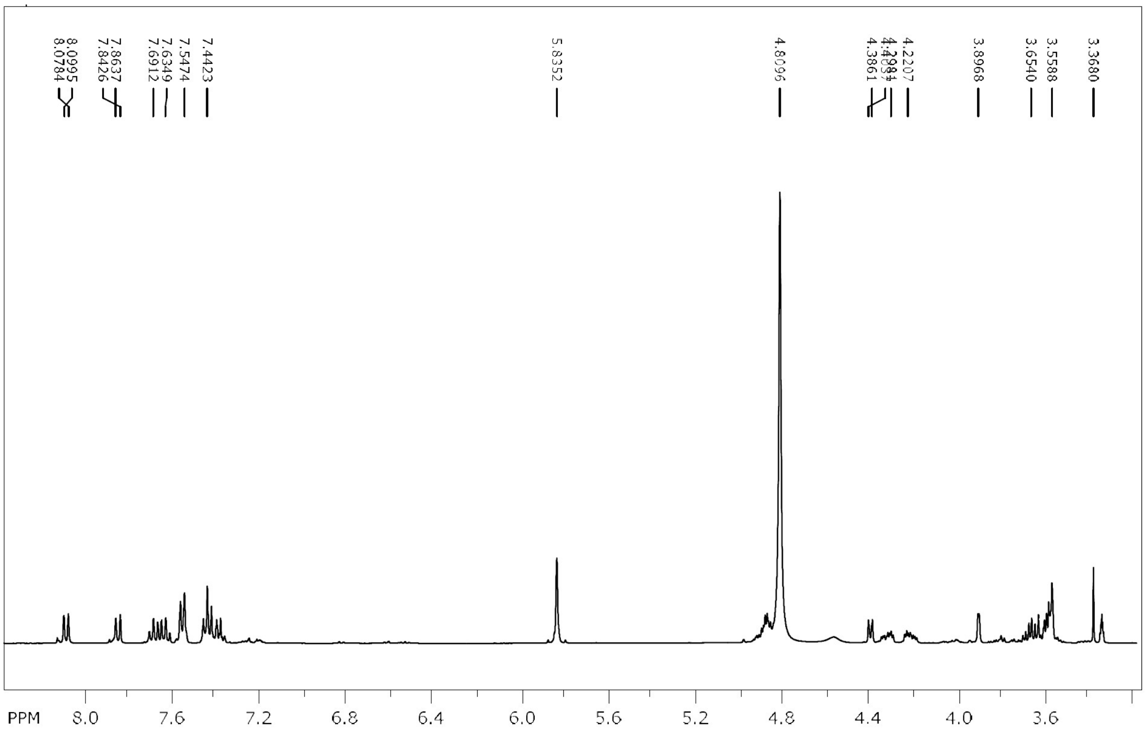

Figure S11. ^{1}H NMR spectra of compound (**14**)

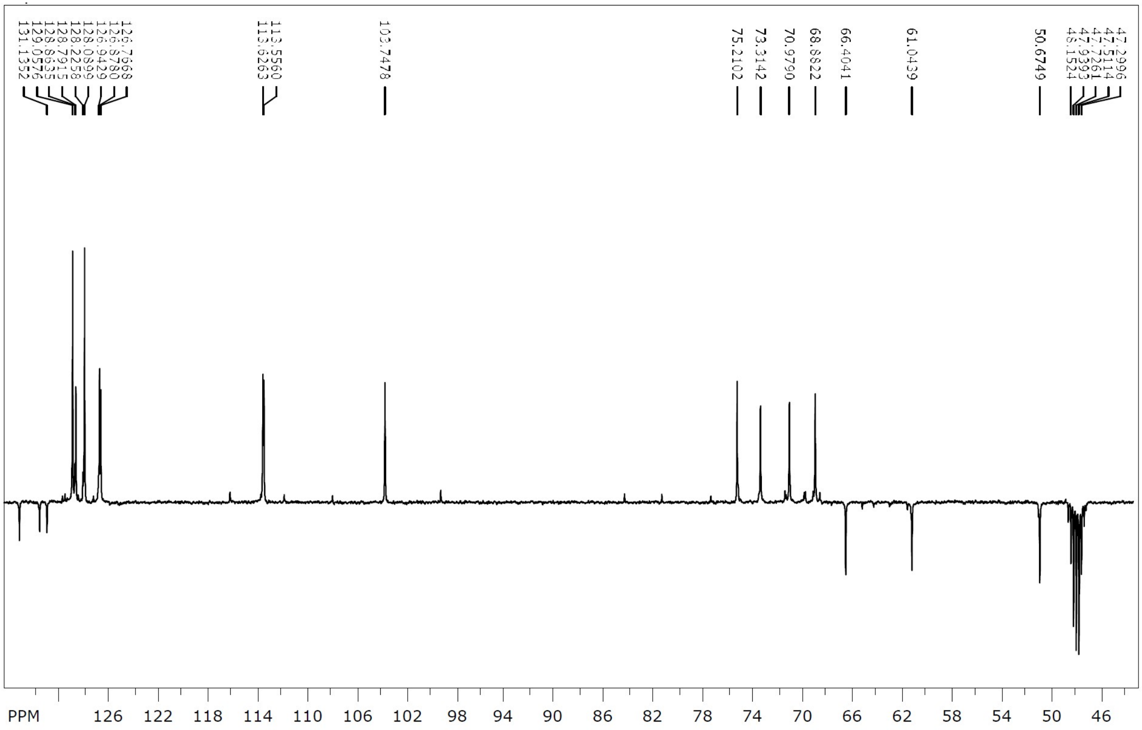

Figure S12. ^{13}C NMR spectra of compound (**14**)
